# Meristic co‐evolution and genomic co‐localization of lateral line scales and vertebrae in Central American cichlid fishes

**DOI:** 10.1002/ece3.70266

**Published:** 2024-09-15

**Authors:** Nicolas Ehemann, Paolo Franchini, Axel Meyer, C. Darrin Hulsey

**Affiliations:** ^1^ Department of Biology University of Konstanz Konstanz Germany; ^2^ Department of Ecological and Biological Sciences Tuscia University Viterbo Italy; ^3^ Museum of Comparative Zoology Harvard University Cambridge Massachusetts USA; ^4^ CAS Key Laboratory of Tropical Marine Bio‐Resources and Ecology South China Sea Institute of Oceanology, Chinese Academy of Sciences Guangzhou China; ^5^ School of Biology and Environmental Science University College Dublin Dublin Ireland

**Keywords:** adaptive radiation, genomic architecture, quantitative genetics, sympatric speciation

## Abstract

Meristic traits are often treated as distinct phenotypes that can be used to differentiate and delineate recently diverged species. For instance, the number of lateral line scales and vertebrae, two traits that vary substantially among Neotropical Heroine cichlid species, have been previously suggested to co‐evolve. These meristic traits could co‐evolve due to shared adaptive, developmental, or genetic factors. If they were found to be genetically or developmentally non‐independent, this might require a more general re‐evaluation of their role in evolutionary or taxonomic studies. We expanded a previous analysis of correlated evolution of meristic traits (lateral line scales and vertebrae counts) in these fishes to include a range of phylogenetic reconstructions as well as the analyses of 13 Nicaraguan Midas cichlid species (*Amphilophus* spp.). Additionally, we performed qualitative traits locus (QTL) mapping in a F2 laboratory‐reared hybrid population from two ecologically divergent Midas cichlid fish species to discover and evaluate whether genomic co‐segregation might explain the observed patterns of meristic co‐evolution. Meristic values for these traits were found to morphologically differentiate some species of the Midas cichlid adaptive radiation. Our QTL analysis pinpointed several genomic regions associated with divergence in these traits and highlighted the potential for genomic co‐segregation of the lateral line and vertebrae numbers on two chromosomes. Further, our phylogenetic comparative analyses consistently recovered a significant positive evolutionary correlation between the counts of lateral line scale and vertebrae numbers in Neotropical cichlids. Hence, the findings of genomic co‐segregation could partially explain the co‐evolution of these two meristic traits in these species. Continuing to unravel the genetic architecture governing meristic divergence helps to better understand both trait correlations and the utility of meristic traits in taxonomic diagnoses and how traits in phenotypes might be expected to co‐evolve.

## Graphical abstract

We dissect the co‐evolution and genetic architecture of lateral line scale counts and vertebrae numbers in Neotropical Heroine cichlids. Our phylogenetic comparative analyses recovered a positive correlation between these two traits. Furthermore, our qualitative traits locus (QTL) analyses identified significant genomic co‐localized associations for upper and lower lateral line scales, caudal vertebrae, total lateral line scales, and total vertebrae. Such shared genomic regions could play important roles in cichlid meristic divergence, as they are expected to bias evolutionary divergence.

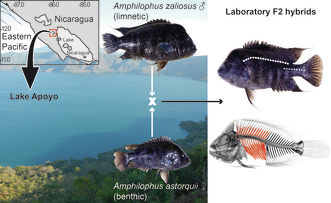

## Resumen

Los rasgos merísticos son empleados comúnmente como caracteres fenotípicos que pueden ser utilizados para diferenciar y delimitar especies recientemente divergentes. Por ejemplo, se ha sugerido anteriormente que el número de escamas de la línea lateral, así como el de vértebras, dos rasgos que varían sustancialmente entre las especies de cíclidos Heroine neotropicales, co‐evolucionan. Estos rasgos podrían co‐evolucionar debido a factores adaptativos, de desarrollo o genéticos compartidos. En el caso de que se descubriera que no son independientes desde el punto de vista genético o del desarrollo, sería necesario reevaluar su función en los estudios evolutivos o taxonómicos. Nosotros hemos ampliado un análisis previo de la evolución correlacionada de los rasgos merísticos (número de escamas de la línea lateral y recuento de vértebras) en estos peces para incluir una serie de reconstrucciones filogenéticas, así como los análisis de las 13 especies de cíclidos nicaragüenses Midas (*Amphilophus* spp.). Además, realizamos un mapeo QTL en una población híbrida F2 criada en laboratorio de dos especies de cíclidos Midas ecológicamente divergentes para descubrir y evaluar si la co‐segregación genómica podría explicar los patrones observados de co‐evolución merística. Se observó que los valores merísticos de estos rasgos diferenciaban morfológicamente algunas especies de la radiación adaptativa de los cíclidos Midas. Nuestro análisis QTL identificó varias regiones genómicas asociadas con la divergencia en estos rasgos y destacó la potencial co‐segregación genómica de la línea lateral y el número de vértebras en dos cromosomas. Además, nuestros análisis filogenéticos comparativos mostraron una correlación evolutiva positiva y significativa entre el número de escamas de la línea lateral y el número de vértebras en los cíclidos neotropicales. Por lo tanto, los hallazgos de co‐segregación genómica podrían explicar parcialmente la co‐evolución de estos dos rasgos merísticos en los cíclidos neotropicales. Seguir desentrañando la arquitectura genética que gobierna la divergencia merística ayuda a comprender mejor tanto las correlaciones de rasgos como la utilidad de los rasgos merísticos en los diagnósticos taxonómicos y cómo podría esperarse que co‐evolucionen los rasgos en los fenotipos.

## 1 INTRODUCTION

Variation in meristic traits, referred to as countable structures occurring in a series, particularly in fishes, is often used to identify, classify, and differentiate species (Armbruster, 2012; Hubbs & Lagler, 1964; Waldman, 2005). Meristic variation could frequently represent adaptative divergence in traits such as body shape, swimming performance, and sensory abilities (Abed et al., 2020; Hermida et al., 2005; Nelson et al., 2016; Sfakianakis et al., 2011). However, different types of meristic traits might not always evolve independently and could even commonly co‐evolve. For instance, traits like the number of lateral line scales and vertebrae numbers distinguish many species, such as sticklebacks, whitefishes, and salmonids (Doherty & McCarthy, 2004; Edge et al., 1991; Haddon & Willis, 1995; Hermida et al., 2005; Hubbs, 1940; Ihssen et al., 1981; Leary et al., 1985). Such meristic variation in these traits has also been linked with differentiation between habitat ecomorphs in Central American Heroine cichlids (Říčan et al., 2016). Divergence in these meristic traits could also be developmentally regulated by shared genes (Bloomquist et al., 2015; Höch et al., 2021). The extent to which these and other phenotypes can evolve independently might commonly be linked to underlying genetic architectures (Albertson et al., 2014; Albertson & Kocher, 2006; Armbruster et al., 2014; Gross et al., 2016; Hulsey et al., 2017, 2019; Kautt et al., 2020; Lande, 1984; Parsons et al., 2016; Wilkens, 2010). Therefore, evolutionary biologists investigating the mechanisms underlying trait co‐evolution have begun to probe the complex interplay among adaptation and physical linkage of genetic loci in the genome (Baumgarten et al., 2015; Conith & Albertson, 2021; Feilich, 2016; Hulsey, Hollingsworth, & Holzman, 2010). Due to the possibility that cichlid fishes' lateral line scales and vertebrae contribute both to habitat adaptation and are frequently used to diagnose species (Barlow & Munsey, 1976; Geiger et al., 2010; Recknagel et al., 2013; Stauffer et al., 2008; Stauffer & McKaye, 2002), we employed phylogenetic comparative analyses and quantitative genetics (QTL mapping) to better understand the genomic basis and potential co‐evolution of these two sets of meristic traits.

**FIGURE 1:**
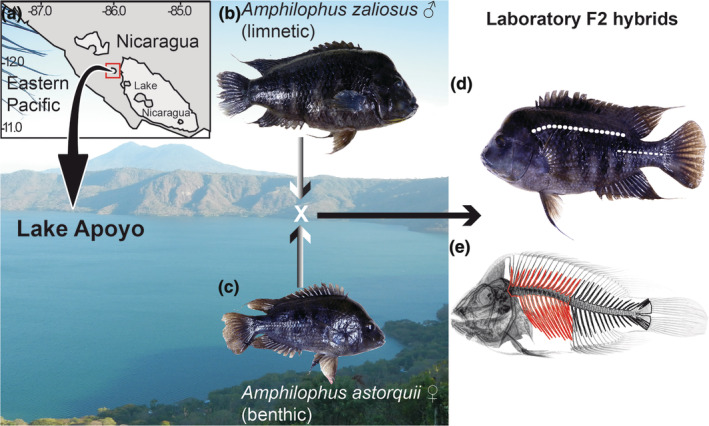
Map of Southwestern Nicaragua (a), indicating the location of crater Lake Apoyo (red box). The limnetic and benthic parent species *Amphilophus zaliosus* (b) and *Amphilophus astorquii* (c) are depicted against a panoramic view of Lake Apoyo, where the parentals were collected in order to obtain the laboratory‐reared F2 hybrids. Midas laboratory‐reared F2 indicating the location of the upper lateral line scales and lower lateral line scales (d). X‐ray image (e) displaying the two regions of the axial skeleton quantified for the vertebrae: Abdominal vertebrae with ribs (red contours) and caudal vertebrae with hemal spines (black contour).

The lateral line system is critical for the perception of external hydrodynamic stimuli in many aquatic vertebrates such as sharks, salamanders, and teleost fishes (Lannoo, 1988; Maruska, 2001; Nelson et al., 2016; Parichy, 1996; Park et al., 2008; Webb et al., 2021). It effectively connects the surrounding aquatic environment with the internal sensory system in all of these lineages of animals and is generally composed of superficial and canal components, including a series of porous scales along the trunk of the body (Bleckmann & Zelick, 2009; Dijkgraaf, 1963; Edgley et al., 2024; Piotrowski & Baker, 2014; Thomas & Raible, 2020; Webb et al., 2021). Many activities, including detection, predator avoidance, social communication, mate choice, conspecific aggression, coordinated swimming such as shoaling, object discrimination, entrainment, rheotaxis, and even navigation in blind cave fishes are sensed by the lateral line (Bleckmann & Zelick, 2009; Coombs et al., 1989; Janssen et al., 1999; Mogdans, 2019; Montgomery et al., 1997; Pitcher, 2012). Furthermore, this system can show extensive variation within and between species, especially with respect to the number and distribution of the scales housing the lateral line's sensory cells. Although the trunk canal neuromast (Bleckmann & Zelick, 2009; Edgley et al., 2024; Webb et al., 2021), referred to as the lateral line scales, forms a continuous line of scales in most teleosts. Species in the Family Cichlidae are distinctive in having an “interrupted” lateral line formed by an anterior upper lateral line and a disjunct, more posterior lower lateral line (Figure 1). Variation in the number of these scales could readily reflect adaptations to different ecological niches and lifestyles, generating a positive correlation between the number of these lateral line scales and flowing water systems (Říčan et al., 2016) or depth preferences (Edgley et al., 2024). However, many investigations have not treated the number of upper and lower lateral line scales as distinct traits (Recknagel et al., 2013; Stiassny & Sparks, 2006; Varella et al., 2023). Whether the number of upper or lower lateral line scales is associated with ecological habitat divergence, such as the benthic vs. limnetic habitat axis, as is common in many lakes (Edgley et al., 2024), is also unclear. Despite the remarkable diversity of the lateral line system in different lineages of teleost fishes, a detailed understanding of the mechanistic underpinnings governing meristic lateral line divergence is still lacking.

The number of vertebrae is another important meristic trait in teleost fishes. Vertebrae numbers have been associated with various environmental factors (Hubbs, 1940; Lindsey, 1975; Maxwell & Wilson, 2013; McDowall, 2008), as well as body elongation, suggesting this variation might often be predictable and adaptive (Aguirre et al., 2019; Ascarrunz et al., 2019; Reyes‐Corral & Aguirre, 2019). Moreover, the addition of vertebrae along the body axis could have a number of functional implications associated with locomotion and maneuverability, including swimming performance and its susceptibility to predators by influencing body flexibility and the ability to curve the body (Baxter et al., 2022; Brainerd & Patek, 1998; Chivers et al., 2008; Kourkouta et al., 2021; Lindsey, 1978; McDowall, 2003; Price et al., 2015; Rouleau et al., 2010; Tibblin et al., 2016). It also may affect sexual selection since in many fish lineages, including cichlids, there exists a tendency for choosing larger individuals to mate with due to their higher fecundity, male–male competition dominance, and resources they can provide to developing offspring (Head et al., 2013; LaManna & Eason, 2011; Passos et al., 2019; Pimentel et al., 2016; Rowland, 1975; Uusi‐Heikkilä, 2020; van denBerghe & Gross, 1989). Because meristic variation in the vertebrae and lateral line influence several of the same organismal functions, such as fish agility, navigation abilities, and overall survival, their co‐evolution might be due solely to shared adaptive divergence rather than mechanistic underpinnings such as shared genetic pathways or homologous genes. Also, because the number of vertebrae fluctuates extensively among diverse groups such as Heroine cichlids, we might expect vertebrae numbers to vary substantially even among very recently diverged cichlid species.

Heroine cichlids represent approximately 20% of Neotropical cichlid species diversity and along with the poecilids and characins they ecologically dominate the Central American fish fauna (Astudillo‐Clavijo et al., 2015; Concheiro‐Pérez et al., 2007; Hulsey et al., 2011; López‐Fernández, 2021; Olvera‐Ríos et al., 2023; Říčan et al., 2016). These cichlids have undergone remarkable diversification, primarily in riverine systems, but they have also diversified extensively in some of the larger lakes in Central America (Hulsey & López‐Fernández, 2011). The Midas cichlid radiation is one such lineage that comprises a recently evolved species complex with 13 currently described species that have diversified only in the past ~20,000 years in a series of Nicaraguan lakes (Barlow, 1976; Barlow & Munsey, 1976; Barluenga et al., 2006; Barluenga & Meyer, 2010; Franchini et al., 2019; Fruciano et al., 2016; Klingenberg et al., 2003; Meyer, 1990a, 1990b; Xiong et al., 2021). Historically, the taxonomy of this group has been complex. For instance, Meek (1907) commented that despite repeated attempts to divide the fish material based on morphological variation, he failed to find a tangible and consistent characteristic to define them resulting from two to half a dozen or more species (Meek, 1907, p. 122). Only since the advent of molecular data has it become possible to resolve the phylogenetics of this species flock across the different Nicaraguan lakes (e.g., Barluenga et al., 2006; Barluenga & Meyer, 2010; Kautt et al., 2020). Midas cichlids are especially notable for their repeated sympatric divergence along the benthic and limnetic habitat axis found in several of these crater lakes (Colombo et al., 2013; Elmer et al., 2010; Franchini et al., 2014, 2019; Fruciano et al., 2016, 2019; Geiger et al., 2010; Kautt et al., 2018, 2020; Meyer, 1990a, 1990b; Recknagel et al., 2013; Říčan et al., 2016). This repeated habitat specialization has been a major driver for the evolution of a number of traits, including cranial morphology, body depth, vision, lip size, teeth, and jaws as well as parallel evolution and hybridization (Barluenga et al., 2006; Colombo et al., 2013; Elmer et al., 2010; Franchini et al., 2014; Fruciano et al., 2016; Kautt et al., 2018, 2020; Klingenberg et al., 2003; Masonick et al., 2023; Olave et al., 2022; Olvera‐Ríos et al., 2023). However, there has been comparatively little investigation into the external or internal meristic traits associated with the divergence in Midas cichlids along the postcranial body axis. For instance, traits such as the number of lateral line scales or vertebrae could readily provide meristic characters that vary and potentially differentiate Midas cichlid species in this adaptive radiation.

Identifying the mechanistic bases of trait correlations has implications for our general understanding of phenotypic evolution (Lande & Arnold, 1983; Sih et al., 2004). Many current models of rapid divergence emphasize the role of genetic linkage and pleiotropy in adaptation and speciation (Archambeault et al., 2020; Hämälä et al., 2020; Noor & Bennett, 2009; Seehausen et al., 2014). For instance, the development of the lateral line system and somites (vertebrae) in African cichlid fishes is governed by over 50 genes exhibiting pleiotropic effects on both traits (Bloomquist et al., 2017). Recent studies have continued to expand the list of pleiotropic genes influencing these structures (Qian et al., 2020; Suzuki et al., 2019; Tang et al., 2019). Trait correlations resulting from pleiotropy are not expected to break down through neutral processes (Chebib & Guillaume, 2021; Jones et al., 2003; Urban et al., 2022). While genetic recombination can erode the linkage between genes subject to selection pressures, strong and persistent correlational selection acting on these genes can counteract this effect and maintain their linkage over time (Saltz et al., 2017; Sinervo & Svensson, 2002; Svensson et al., 2021). Trait correlations generated by genetic linkage or pleiotropy (Mauro & Ghalambor, 2020; Papakostas et al., 2014) could, therefore, often be transient and contribute less to evolutionary change (Saltz et al., 2017). Nevertheless, if numerous qualitative traits locus (QTL) were shared between meristic traits like the number of lateral line scales and the number of vertebrae, this might explain substantial co‐evolution of these traits in radiations such as the Heroine cichlids.

To investigate the meristic co‐evolution of lateral line scales and vertebrae in Heroine cichlids, we quantified the number of upper and lower lateral line scales as well as the abdominal and caudal vertebrae in a number of wild‐caught and laboratory‐raised Midas cichlid fishes. We first tested the utility of these traits for distinguishing among all Nicaraguan Midas species. Then, using multiple phylogenetic reconstructions and total meristic counts for both vertebrae and lateral line scales of Heroine cichlids, we examined the macroevolutionary correlations between these traits across 86 of the ~160 species comprising Heroine cichlid diversity. Finally, we explored the genetic underpinnings of these meristic traits using a laboratory‐reared Midas F2 hybrid mapping population of two sympatric and endemic Nicaraguan crater Lake Apoyo *Amphilophus* species. These two species are divergent along the lake's benthic and limnetic axis and exhibit meristic variation in several of our traits of interest. By combining these analyses, we asked whether, and to what extent, genome co‐segregation could have contributed to cichlid lateral line and vertebrae meristic co‐evolution.

## 2 METHODS

### 2.1 Lateral line and vertebrae count

Meristic variation in 371 Midas cichlid individuals was quantified (Table 1). We examined both the number of lateral line scales and vertebrae for these fishes. For our analyses, 173 wild‐caught Nicaraguan Midas specimens taken from the 13 currently named Midas cichlids species in the *Amphilophus* species complex were examined (Table S1). The meristic data, including lateral line scales and vertebrae numbers, were gathered from specimens of all Nicaraguan Midas species collected in the two Nicaraguan Great Lakes (Lake Managua and Lake Nicaragua) and several nearby crater lakes (Figure 1a, Table 1). Since the Midas cichlid radiation is a recently evolved group, its taxonomy can be complex. Fortunately, all the specimens used here for the collection of our meristic data were recently examined by Kautt et al. (2020) via whole genome sequencing. Therefore, our taxonomic determinations are based on these previous genome‐level assessments. Additionally, we quantified meristic variation in 198 laboratory‐reared second‐generation (F2) hybrids from an interspecific cross that were raised and genotyped for QTL analyses and have been used in previous studies (Franchini et al., 2014; Fruciano et al., 2016; Kautt et al., 2020). These F2 hybrid individuals were formed by interbreeding two Lake Apoyo endemic species, *Amphilophus zaliosus* (Figure 1b) and *Amphilophus astorquii* (Figure 1c).

**TABLE 1:** The number of lateral line scales and vertebrae in Midas cichlid species and in Lake Apoyo F2 hybrids were quantified.

Species	*n*	Lake	Microhabitat	Lateral line scales			Abdominal	Vertebrae	Total
				Upper	Lower	Total		Caudal	
*A. amarillo*	16	Xiloá	Benthic	18–23 (21.1)	10–13 (10.8)^abcd^	28–34 (31.9)	14–14 (14.0)	15–17 (15.9)	29–31 (29.9)
*A. chancho*	02	Apoyo	Benthic	21–21 (21.0)	13–14 (13.5)	34–35 (34.5)	14–14 (14.0)	15–16 (15.5)	29–30 (29.5)
*A. citrinellus*	17	AsL	Benthic	19–22 (21.0)	10–14 (12.1)^aef^	31–35 (33.1)^§^	14–14 (14.0)	14–17 (15.9)	28–31 (29.9)
*A. citrinellus*	17	Apoyeque	Benthic	18–22 (20.5)*	09–12 (11.4)^g^	30–34 (31.8)^†^	14–14 (14.0)	15–16 (15.9)	29–30 (29.9)
*A. citrinellus*	14	GLM	Benthic	19–22 (20.9)	10–14 (12.1)^bhi^	31–36 (32.9)^‡^	14–14 (14.0)	15–16 (15.9)	29–30 (29.9)
*A. citrinellus*	04	GLN	Benthic	20–22 (21.0)	12–13 (12.2)	32–34 (33.2)	14–14 (14.0)	16–16 (16.0)	30–30 (30.0)
*A. citrinellus*	07	Masaya	Benthic	20–24 (21.4)	11–14 (12.1)	31–38 (33.6)	14–14 (14.0)	15–16 (15.4)	29–30 (29.4)
*A. citrinellus*	16	Tiscapa	Benthic	19–23 (21.1)	11–13 (12.2)^cjk^	32–34 (33.2)^#^	14–14 (14.0)	15–16 (15.9)	29–30 (29.9)
*A. flaveolus*	04	Apoyo	Limnetic	21–23 (21.5)	10–11 (10.8)	31–34 (32.3)	14–14 (14.0)	15–16 (15.5)	29–30 (29.8)
*A. globosus*	05	Apoyo	Benthic	20–23 (21.4)	11–13 (11.6)	31–35 (33.0)	14–15 (14.8)	14–16 (15.0)	29–31 (29.8)
*A. labiatus*	12	GLM	Benthic	20–22 (21.2)	11–12 (11.8)^lm^	32–34 (32.9)^††^	14–14 (14.0)	15–16 (15.9)	29–30 (29.9)
*A. sagittae*	22	Xiloá	Limnetic	18–24 (21.6)*	09–12 (10.6)^ehjln^	28–35 (32.2)	14–14 (14.0)	15–16 (15.9)	29–30 (29.9)
*A. supercilius*	01	Apoyo	Limnetic	19–19 (19.0)	11–11 (11.0)	30–30 (30.0)	14–14 (14.0)	16–16 (16.0)	30–30 (30.0)
*A. tolteca*	20	AM	Limnetic	20–23 (21.2)	10–13 (11.8)^dno^	30–36 (33.1)*	14–15 (14.0)	14–16 (15.7)	28–31 (29.8)
*A. viridis*	04	Xiloá	Benthic	21–24 (22.6)	11–12 (11.3)	33–35 (34.0)	14–14 (14.0)	15–16 (15.3)	29–30 (29.3)
*A. xiloaensis*	08	Xiloá	Benthic	19–22 (20.7)	09–11 (10.1)^fgikmo^	30–32 (30.9)^§ †‡#††*^	14–14 (14.0)	15–16 (15.7)	29–30 (29.6)
*A. astorquii*	02	Apoyo	Benthic	19–23 (21.0)	08–12 (10.0)	27–35 (31.0)	14–14 (14.0)	16–16 (16.0)	30–30 (30.0)
*A. zaliosus*	02	Apoyo	Limnetic	22–22 (22.0)	12–12 (12.0)	34–34 (34.0)	14–14 (14.0)	16–16 (16.0)	30–30 (30.0)
F2 hybrids	198			08–22 (19.0)	00–14 (10.2)	15–27 (29.2)	14–15 (14.0)	15–17 (16.1)	29–31 (30.1)

*Note*: Meristic minimum to maximum as well as (mean) values of the lateral line scales (upper, lower, and total) and vertebrae (abdominal, caudal, and total) in 13 Nicaraguan *Amphilophus* spp., six populations of *A*. *citrinellus* (indicated by the lake of collection), as well as the laboratory‐reared F2 hybrids generated between *A. astorquii* and *A. zaliosus* were assessed. The individual Nicaraguan lakes where these wild‐caught specimens were collected for the population comparisons included Apoyeque, Apoyo, Asososca Managua (AM), Asososca León (AsL), Great Lake Managua (GLM), Great Lake Nicaragua (GLN), Masaya, Tiscapa, and Xiloá. Benthic microhabitat refers to whether species generally forage on the bottom, and limnetic refers to foraging in the water column (Kautt et al., 2020). A post hoc test (*p* < .05) was performed on the ANOVA results for multiple comparisons of the meristic values for the species having more than eight specimens, and differences are highlighted with superscript letters and symbols. Vertebrae numbers were not found to differ significantly among the Midas cichlids. Upper lateral line scales only differed between the two groups (*). However, lower lateral line scale numbers differed in several ways among the Midas species and populations (^a–o^), and the total lateral line scales did differ between six *Amphilophus* lineages (^†§‡#††*^).

To quantify the meristic variation in the lateral line, the number of scales in both the upper and lower lateral lines (Figure 1d) were counted on the left side of the body of each Midas specimen. The total lateral line scales, taken as the sum of these two traits were also tabulated. To visualize the Midas cichlids vertebrae numbers, we used x‐rays similar to methods employed previously (Aguirre et al., 2016; Chen et al., 2020; Dayal et al., 2022; Ehemann et al., 2024; Reyes‐Corral & Aguirre, 2019). To obtain the best possible X‐ray resolution of the vertebrae, we adjusted the configuration of the X‐Ray ZooMax Gold machine (Control X Medical, Budapest, Hungary) to 52,000 kilovoltage and 10.0 milli‐amperes/second. The total counts of the vertebrae were divided into abdominal vertebrae, characterized by bearing the ribs, and caudal vertebrae, which have only hemal spines (Figure 1e).

We estimated the range and mean values of the different lateral line scales and vertebrae numbers for each Midas species, including meristic values at a population level of *A*. *citrinellus* from different lakes, as well as for the laboratory‐reared F2 hybrids. Importantly, the number of lateral line scales is defined during early larval development and is not expected to change as the fish grows (Ghysen & Dambly‐Chaudière, 2004; Ghysen et al., 2014; Wada & Kawakami, 2015). Similarly, the number of vertebrae does not change during ontogeny (Ando et al., 2008; Darias et al., 2011; Nikiforidou et al., 2020), where cichlids fishes and particularly *Amphilophus* species attain an adult body plan between 2 and 4 weeks after fertilization (Molina‐Arias, 2011; Woltering et al., 2018). Therefore, no size correction for these meristic traits was implemented. Additionally, a principal component analysis (PCA) was performed using the data gathered here for the upper and lower lateral line scales as well as for the abdominal and caudal vertebrae to visualize the Midas group's distribution in this morphospace.

### 2.2 Phylogenetic comparative analyses

Mean values of the number of vertebrae and lateral line scales were taken for 73 Heroine species previously published by Říčan et al. (2016). In addition, we added comparative data spanning between one and 22 wild‐caught specimens of all 13 described Midas cichlid species. To avoid bias in our phylogenetic comparative analyses due to mixing meristic values from different populations, we exclusively considered *A*. *citrinellus* data obtained from the individuals collected in the Great Lake Nicaragua, which corresponds to the type locality of this species (Günther, 1864). Species taxonomy of the Heroine species included in our analyses was updated according to the online Catalog of Fishes database (Fricke et al., 2023).

For the phylogenetic comparative analyses, we followed a methodological approach similar to Hulsey and Gorman (2023) in order to incorporate phylogenetic uncertainty into our tests of the phylogenetic correlation of the number of lateral line scales and vertebrae. Our phylogenetic estimation of Heroine cichlid relationships was based on combinations of three different phylogenetic datasets: (i) a cytochrome b (*cytb*) mitochondrial gene tree (Hulsey, Hollingsworth, & Fordyce, 2010), (ii) ddRADseq (Říčan et al., 2016), and (iii) a Midas cichlid whole‐genome phylogeny (Kautt et al., 2020). The *cytb* and ddRADseq phylogenetic reconstructions represent some of the most complete species‐level sampled phylogenies for this group and served as the backbone tree, but neither originally included a substantial number of Midas cichlid species, nor is the molecular variation sufficient to result in strongly supported phylogenetic hypotheses. Therefore, we combined the Midas cichlid resequenced genome phylogeny (Kautt et al., 2020) with both the *cytb* phylogeny and separately with the ddRADseq phylogeny using the function “tree. merger” implemented in the R package “RRphylo” version 2.8.0 (Castiglione et al., 2018) within the R programming environment (R Core Team, 2022). To render the branch lengths ultrametric, we first employed nonparametric rate smoothing based on penalized likelihood (Sanderson, 2002) using the function “chronos” with a lambda smoothing parameter of 0.2. This lambda value represents a trade‐off between each branch having its own rate (lambda = 0.0) and increased lambda values where similar rate variation is assumed for all branches. We then calculated the phylogenetic independent contrasts (pic) for the mean values of lateral line and vertebrae numbers among the Heroine species and examined their correlation using “ape” version 5.7‐1 package (Paradis & Schliep, 2019). To further account for phylogenetic uncertainty in the recovered topology and branch lengths for the independent contrast analyses, we also performed 100 permutations of relationships and branch lengths using the “swapONE” function implemented in the R package “RRphylo” (Castiglione et al., 2018). These 100 randomizations and a switching node position probability set to 50% allowed us to produce standard errors associated with the phylogenetically corrected correlation coefficients and the associated *p*‐values of the pic correlations.

### 2.3 Qualitative traits locus (QTL) mapping

We utilized a previously generated laboratory‐reared F2 hybrid population involving two ecologically distinct yet closely related Midas species, the limnetic *A. zaliosus* (Figure 1b) and the benthic *A. astorquii* (Figure 1c). The parent species differ in body shape, with *A*. *zaliosus* being more elongated and laterally compressed (Elmer et al., 2010; Franchini et al., 2014). Furthermore, these F2 hybrids born of a single pair of parents generally exhibited a mixture of morphological traits found in both parents (Franchini et al., 2014). Meristic traits had not been assessed in these specimens before. We quantified the number of upper lateral line scales, lower lateral line scales, total lateral line scales, abdominal vertebrae, caudal vertebrae, and total vertebrae number of 198 individuals (Table 1) and performed QTL mapping with this hybrid population to identify the genomic regions underlying the relevant morphological traits assessed (Table S2).

Further details regarding the F2 laboratory‐reared hybrids and the methodological description of the QTL genotyping and analyses used here can be found in our previous publications (Franchini et al., 2014; Fruciano et al., 2016). But, in brief, the linkage map was constructed with markers obtained through ddRADseq for the F2 hybrid individuals exclusively for the samples that also had the meristic data. Simultaneously, markers from the two parental specimens were derived from a combination of ddRADseq and genome resequencing. Quality‐controlled sequences were aligned to the chromosome‐level genome assembly of *Amphilophus citrinellus* (NCBI accession number: GCA_013435755.1). Only markers meeting specific criteria were retained to ensure the linkage map's accuracy. These criteria included markers for which both parents were homozygous for different alleles or only one of the parents was heterozygous, and for F2 genotype frequencies conformed to the expected segregation ratio (evaluated through an *X* ^2^ test with a significant threshold of *p* = .001). Individuals with at least 90% of their genotypes successfully called and loci present in at least 80% of the individuals were included. This stringent approach was employed to guarantee the quality and reliability of the linkage map.

The final linkage map was constructed using a regression‐based algorithm that implemented the Kosambi mapping function. This map was originally created using data from 279 F2 individuals and 594 markers that spanned 24 linkage groups (LGs) corresponding to the 24 chromosomes of the Midas cichlid (Kautt et al., 2020). Next, using a Haley‐Knott regression (Haley & Knott, 1992), the number of upper, lower, and total lateral line scales along with the abdominal, caudal, and total vertebrae were mapped independently onto the LGs in the R package R/qtl version 1.60 (Broman et al., 2003). The logarithm of the odds (LOD) threshold for testing the significance of the QTL peaks was calculated using 1,000 permutations for each trait, and the significance for either genome‐wide or for each LG/chromosome was estimated. We applied a suggestive chromosome‐level threshold (*p* = .1) for each trait to identify candidate QTL loci. These loci were then incorporated into a multiple QTL model using the *fitqtl* function within the R/qtl package. Multiple regression *F*‐tests (*p* = .05) were employed to evaluate the models. Nonsignificant QTLs were systematically removed during model selection, which allowed the refinement and optimization of the QTL model. Each QTL's position and LOD score were further refined using the R/qtl *refineqtl* function. This iterative approach ensured the accuracy of the QTL positions and their associated LOD scores. To test scenarios of overlapping QTL among the meristic traits, each QTL's peak, including left and right boundaries along with the maximum LOD score, was calculated using a 95% Bayesian credible interval using the *bayesint* function within the R/qtl package.

Finally, we attempted to identify putative candidate loci located underneath the QTL peaks in the Midas cichlid genome. We explored candidate loci shown to be expressed in the lateral line and vertebrae in Bloomquist et al. (2017), which provided a compendium for developmental gene expression in African cichlids. Then, we identified the location of all of these genes involved in somite and lateral line sensory system development that were also located under our significant QTL peaks based on the Midas genome assembly (Kautt et al., 2020).

## 3 RESULTS

### 3.1 Lateral line and vertebrae count

In our examination of wild‐caught Midas species, we observed a range of variation in the upper lateral line scales from 18 to 24 scales. Among these, the benthic *A. viridis* exhibited the highest mean value of 22.6 upper lateral line scales, while the limnetic species *A. supercilius* displayed the lowest mean value of 19.0 scales (Table 1). However, statistical differences for this trait among the Midas groups having at least eight individuals (*F*_(8,133)_ = 1.82, *p* = .05) were present exclusively between the benthic species *A. citrinellus* of the Apoyeque crater lake population and the limnetic *A*. *sagittae* in the Xiloá crater lake (Table 1). These two species had mean upper lateral line scale values of 20.5 and 21.6, respectively (Table 1).

For the lower lateral line scales, we observed that the benthic species *A. astorquii* presented the minimum value of eight scales, while the two benthic species *A. chancho* and *A*. *citrinellus* (Asososca León, Great Lake Managua, and Masaya populations) exhibited the maximum number of 14 scales (Table 1). The mean counts for lower lateral line scales across the Midas species ranged from 13.5 (*A*. *chancho*) to 10.0 in *A*. *astorquii* (Table 1). There were statistical differences among these and several of the Midas species evaluated (*F*_(8,133)_ = 11.27, *p* < .001). For example, the benthic species *A*. *xiloaensis* had the lowest lateral line scale counts (10.1), significantly different from the ≥12.1 estimated for several *A*. *citrinellus* populations and the 11.8 recorded for another limnetic Midas species, *A*. *tolteca*, as well as the benthic *A*. *labiatus* (Table 1). Similarly, the benthic species *A*. *amarillo* (10.8) differed statistically from several *A*. *citrinellus* populations and *A*. *tolteca* (Table 1).

In terms of the total lateral line scales, the numbers varied from a low of 27 in *A*. *astorquii* to a high of 38 in *A*. *citrinellus* Masaya's population. The limnetic species *A*. *zaliosus* displayed one of the highest mean values (34.0), while the benthic *A. astorquii* exhibited one of the lowest mean values at 31.0 for total lateral line scales (Table 1). Significant differences were found for this trait among the Midas groups assessed (*F*_(8,133)_ = 4.53, *p* < .001). For instance, the mean for this trait was statistically lower in the benthic species *A*. *xiloaensis* (30.9) when compared to the other five Midas groups, such as *A*. *citrinellus* in Asososca León (33.1) and *A*. *labiatus* (32.9), as well as for *A*. *citrinellus* in Apoyo and *A*. *citrinellus* in Tiscapa (Table 1). However, there was no unambiguous association across the Midas radiation between lake microhabitat specialization and lateral line scale counts.

In the wild‐caught Midas cichlids examined, the abdominal vertebrae showed only slight variation ranging from 14 to 15 vertebrae. The most common count among species was 14 vertebrae, except for the benthic species *A. globosus*, which exhibited a higher mean value of 14.8 (Table 1). In contrast, a broader range of variation was observed in the caudal region, where the counts spanned from 14 to 17 vertebrae (Table 1). Interestingly, the benthic *A. astorquii*, along with the limnetic species *A. zaliosus* and *A*. *supercilius*, were found to have the highest mean values for caudal vertebrae at 16.0, and *A. globosus* displayed the lowest mean value of 15.0. A similar pattern was also observed for the mean total vertebrae number, which ranged from 29.5 in *A*. *chancho*, a benthic species, to 30.0 in the species *A*. *astorquii*, *A*. *superercilius*, and *A*. *zaliosus*, the latter two species having a more limnetic microhabitat preference. However, no significant differences (*F*_(8,133)_ = 2.03, *p* > .05) were estimated for any of the vertebrae counts among the Nicaraguan species compared (Table 1).

**FIGURE 2:**
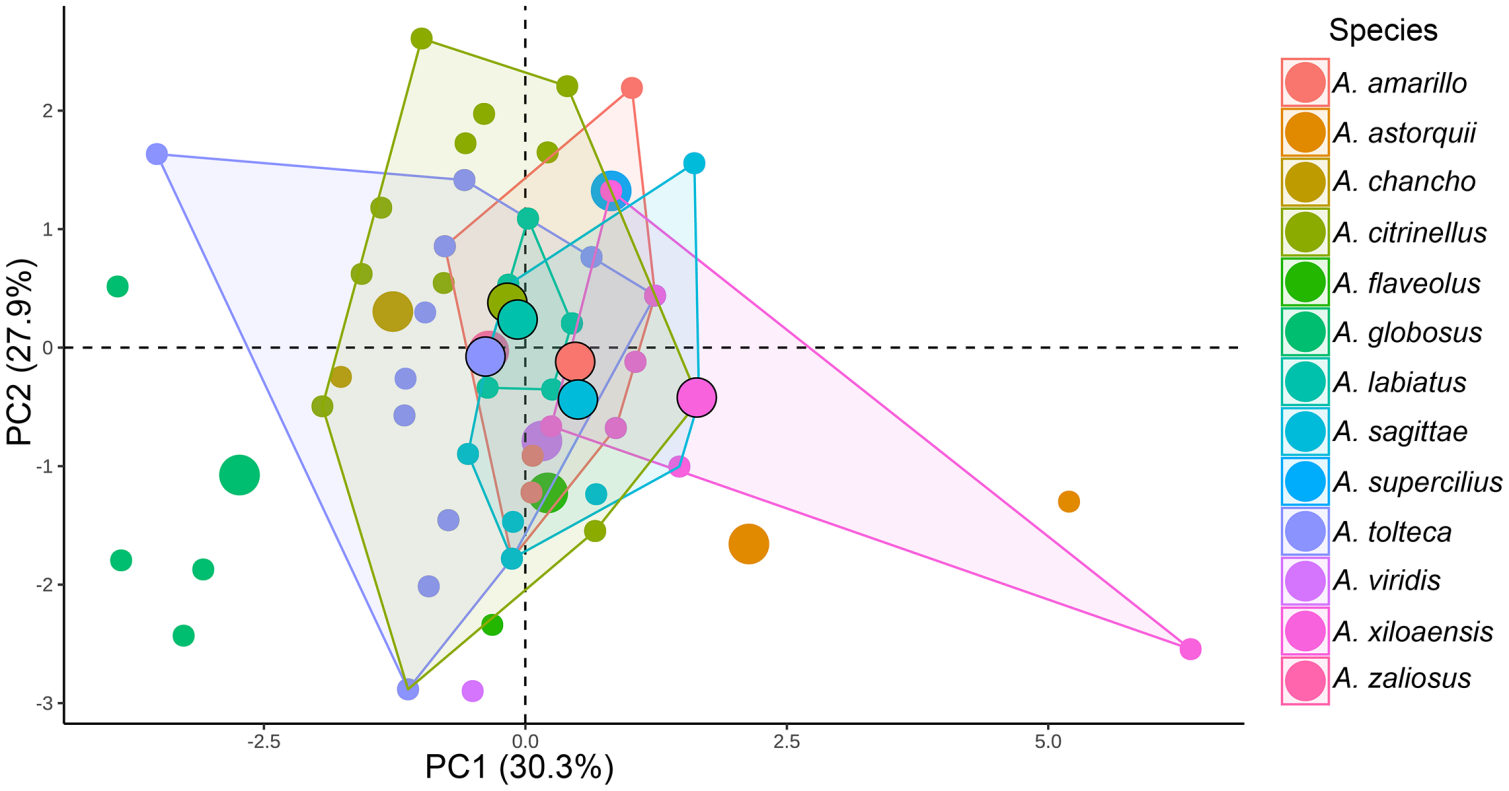
Principal Component Analysis of the upper and lower lateral line scales as well as the abdominal and caudal vertebrae numbers for the 13 Midas species examined. Each color corresponds to a distinct *Amphilophus* species, where the more prominent circle symbols indicate its centroid value. The PC1 is negatively correlated to the abdominal vertebrae trait. Meanwhile, PC2 positively correlates with caudal vertebrae and lower lateral line scales. The convex hull is shown for the groups with at least eight individuals subsequently used for the ANOVA multi‐comparison tests detailed in Table 1.

Finally, our two first PC axes, based on our Midas upper and lower lateral line scales, as well as for the abdominal and caudal vertebrae data, explained 58.2% of the total variation (Figure 2). The PC1 (30.3%) was negatively correlated to abdominal vertebrae and lower and upper lateral line scales. In general, PC2 (27.9%) was positively correlated with caudal vertebrae and lower lateral line scales (Figure 2). Despite the overlapping areas among the Midas groups due to the similarities in their meristic data, *A*. *globosus* occupied a distinct region of morphospace in the PCA (Figure 2). Regardless of the collection site, the benthic species *A*. *citrinellus* and *A*. *labiatus* were grouped closely together (Figure 2).

### 3.2 Phylogenetic comparative analyses

**FIGURE 3:**
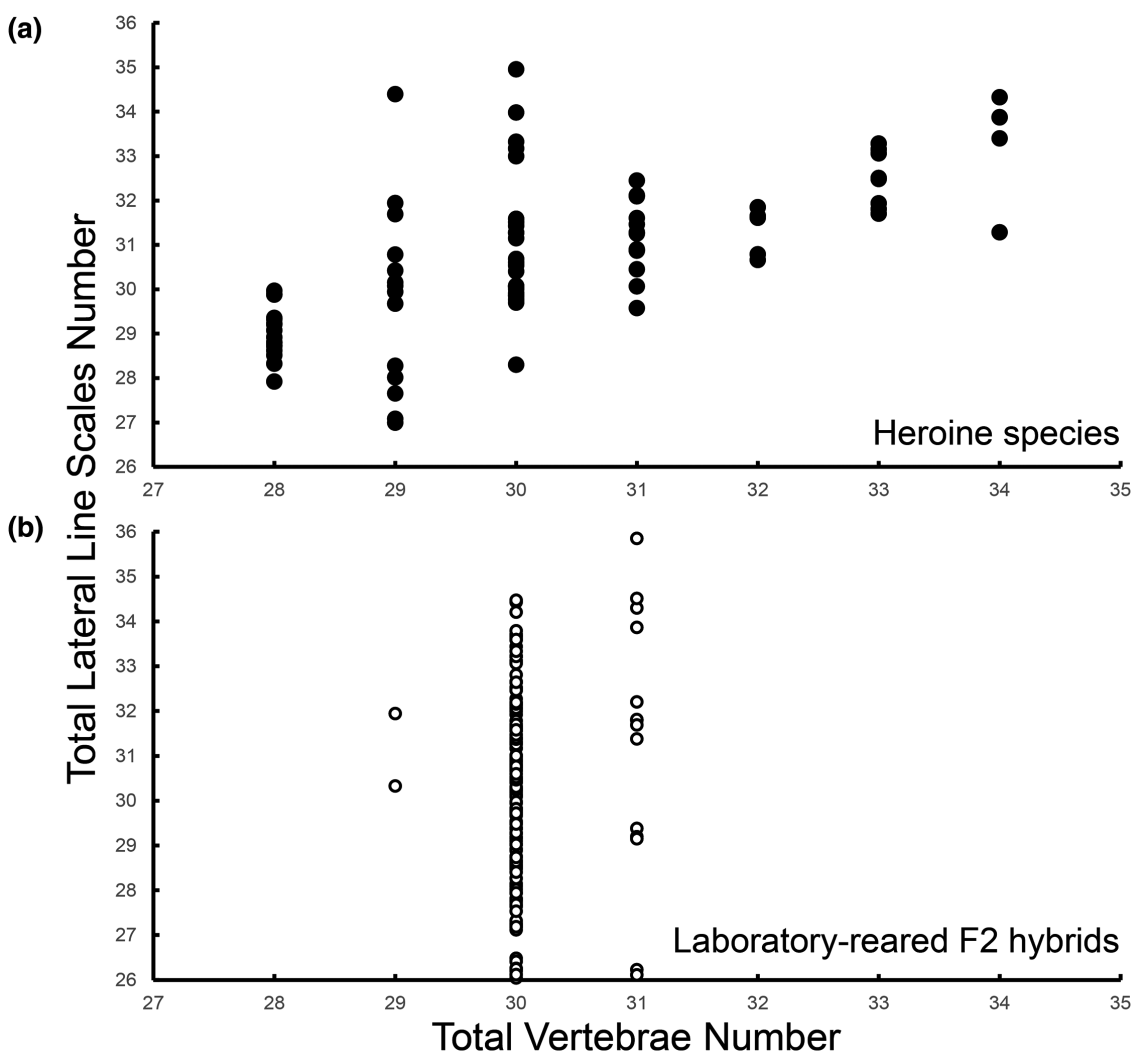
Plot showing the number of the total lateral line scales and total vertebrae number recorded for the Neotropical Heroine cichlids species (a) where each filled circle represents a species. The general trend across species of an increase in total lateral line scales in association with an increase in vertebrae numbers is evident. Statistical correlation analyses on these Heroine species values were not performed because the relationship is not corrected for the phylogenetic history. The total number of lateral line scales and vertebrae is also shown for our laboratory‐reared F2 hybrids (b).

**FIGURE 4:**
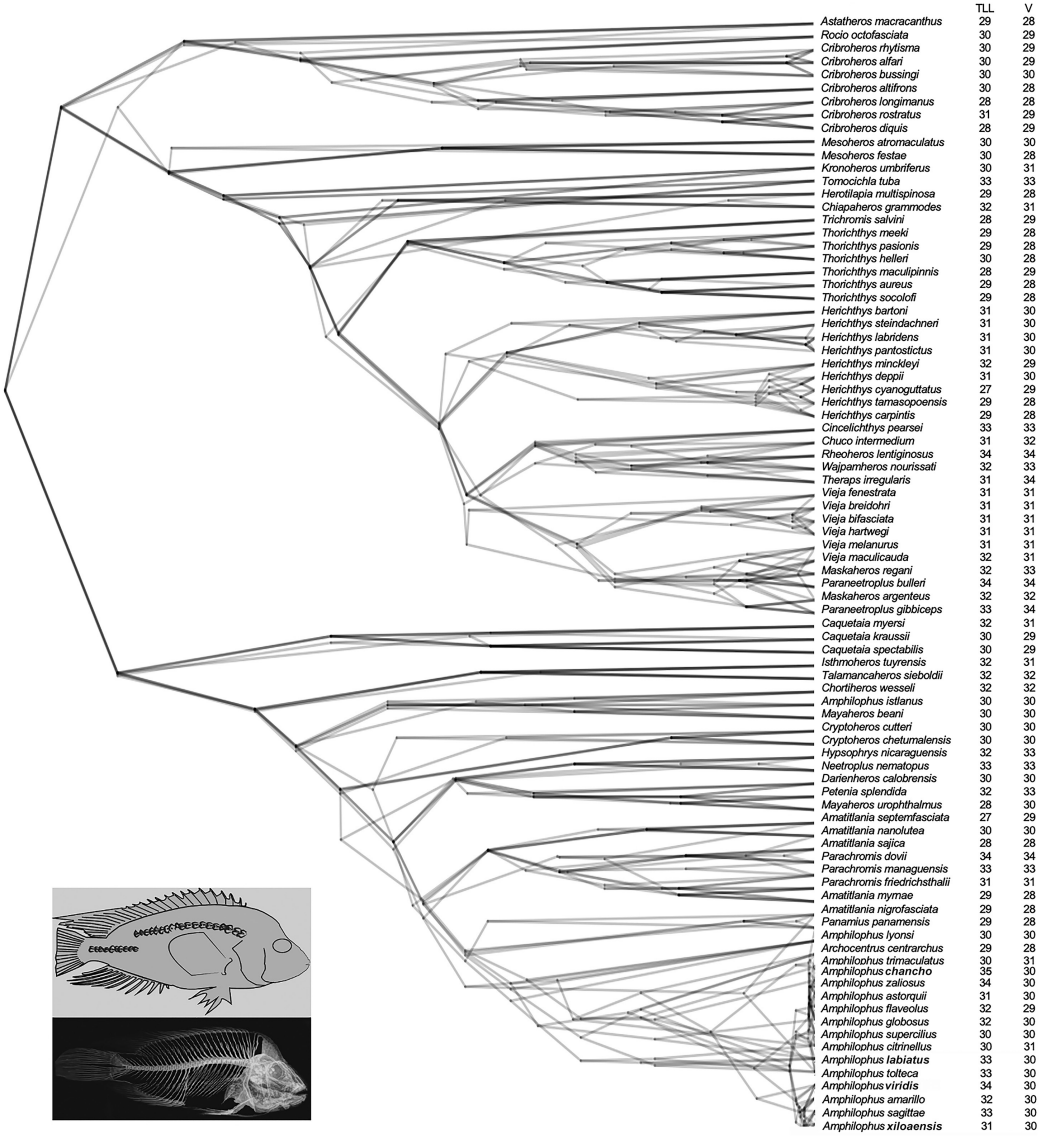
Phylogenetic densitree depicting the mean values of total lateral line scales (TLL) and total vertebrae (V) for 86 species of Neotropical Heroine cichlids. The potential variability in the comparative phylogenetic framework of the cichlid species is represented by five of the 100 topological permutations of the merged cytochrome b mitochondrial phylogeny from Hulsey, Hollingsworth, and Fordyce (2010) and the whole genome phylogeny from Kautt et al. (2020). Species mean meristic values were taken from Říčan et al. (2016) and Table 1.

Our phylogenetic comparative analysis incorporated data on the mean values for the total lateral line scales and total vertebrae counts across 86 Neotropical Heroine species, where the data of 73 Heroine species were taken from Říčan et al. (2016), and the remaining 13 belong to the *Amphilophus* spp. meristic information gathered here (Figure 3a). Within the Heroine group, there was substantial variation in the total lateral line scales spanning from 27 to 35 (Figure 3a), with an overall inter‐species average of 30.8. Similarly, the total vertebrae counts exhibited notable variation, ranging from 28 to 34 (Figure 3a), with an average within the Heroine group species of 30.2 vertebrae. Less variation was present in our 198 laboratory‐reared F2 hybrids dataset (Figure 3b). Based on the Heroine meristic dataset and using 100 randomizations for phylogenetically corrected correlation coefficient calculation, a significant positive phylogenetic correlation was estimated between the total lateral line scales and total number of vertebrae (*cytb*: *p* = .034 ± .084, *r* = .289 ± .117; ddRADseq: *p* = .014 ± .024, *r* = .462 ± .120). These results indicate a relatively robust co‐evolutionary trend, where changes in these meristic traits tend to occur in concert among closely related Heroine species (Figure 4).

### 3.3 Qualitative traits locus (QTL) mapping

**FIGURE 5:**
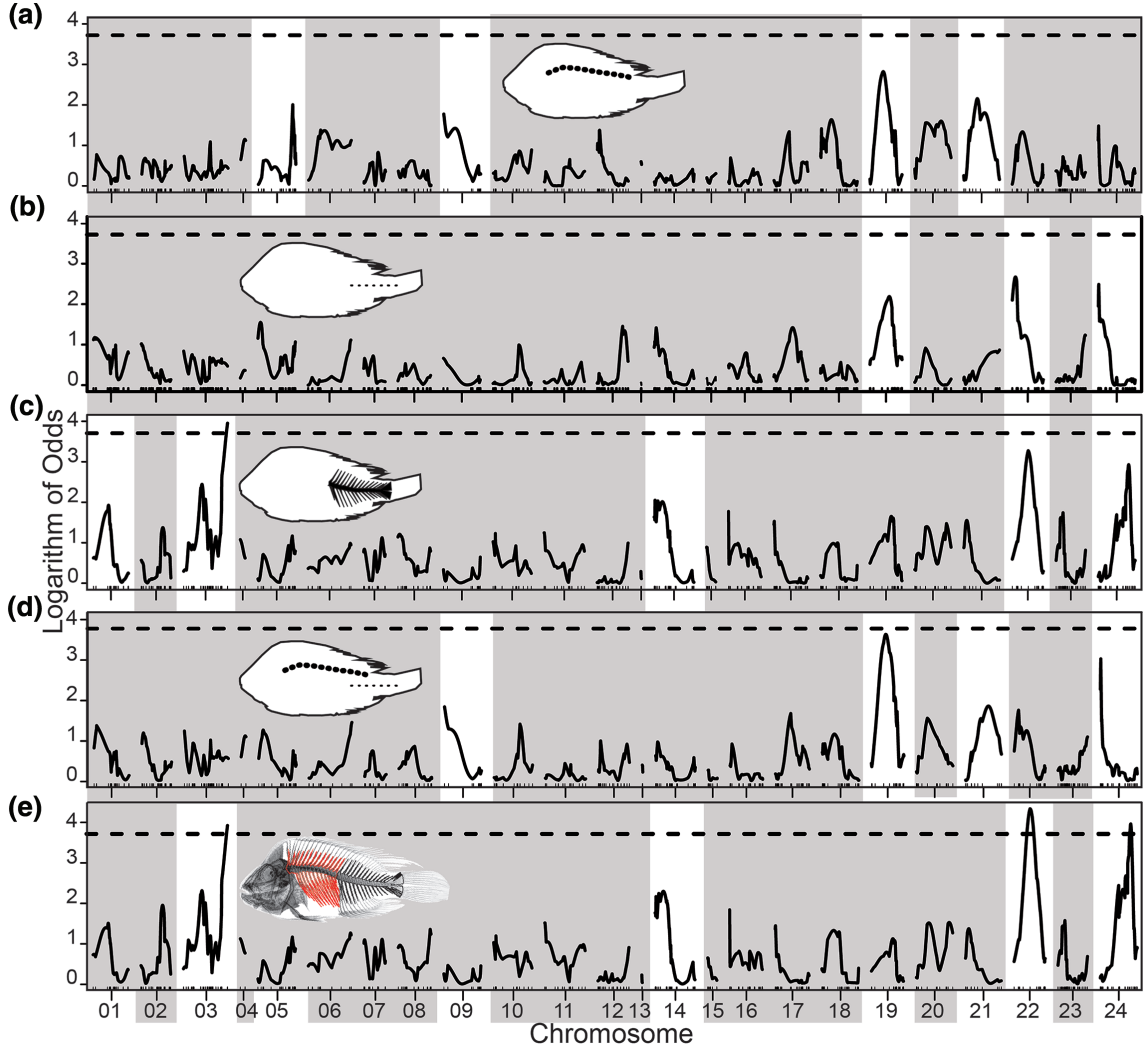
Quantitative Trait Locus (QTL) analyses of the meristic traits. The LOD scores at each linkage group (LG) that generally correspond to chromosomes are shown for the upper lateral line scales (a), lower lateral line scales (b), caudal vertebrae (c), total lateral line scales (d), and total vertebrae (e). The latter two traits correspond to a cumulative effect of the variation accounted for the combined upper and lower lateral line scales as well as for the combined abdominal and caudal vertebrae numbers, respectively. The horizontal dotted black line identifies the genome‐wide significance threshold obtained through 1000 permutations. White vertical bars running through the QTL plots highlight the linkage groups with significant QTL (*p* < .05) for each trait, and highlight the co‐localized QTL regions identified in this study among the different traits.

The QTL mapping analysis of lateral line and vertebrae counts was conducted on 187 F2 laboratory‐reared specimens since 12 of the initially 198 meristically assessed individuals were not successfully genotyped. Our QTL explained a certain amount of the variation, however, the unexplained portion could be related to plasticity or genetic effects such as loci of small‐effect and epistasis not detected in our analysis. Nevertheless, our results revealed statistically supported QTL for the upper lateral line scales (Figure 5a), lower lateral line scales (Figure 5b), caudal vertebrae (Figure 5c), total lateral line scales (Figure 5d), and total vertebrae (Figure 5e). The latter two represented as a cumulative effect of the meristic variation accounted for the upper and lower as well as for the abdominal and caudal vertebrae traits, respectively. Interestingly, our laboratory‐reared F2 hybrids exhibited mean upper (19.0), lower (10.2), and total (29.2) lateral line scales that were comparable to most of the Midas species but with a substantial range when compared to individual wild‐caught Midas species (Table 1). In addition, these F2 hybrids had mean counts of 14.0 abdominal, 16.1 caudal, and 30.1 total vertebrae, consistent and closely aligned with the values recorded for the wild Midas species (Table 1).

The drop one QTL ANOVA revealed several LGs associated with variation in the lateral line and vertebrae counts (Table 2). Specifically, the model indicated a genetic basis for the upper lateral line scales, with four QTLs explaining 18.6% of the variation (Table 2). Similarly, the lower lateral line scales showed three significant QTLs, accounting for about 15.7% of the variance (Table 2). The model suggested four QTL LGs for the total lateral line scales, explaining 21.0% of the variation (Table 2). Interestingly, the QTL on LGs 9, 19, 21, and 24 were identified as potentially important for two or more of the previously described traits (Table 2).

**TABLE 2:** Quantitative genetic analyses of lateral line scale numbers and vertebrae numbers in the laboratory‐reared F2 Lake Apoyo hybrid mapping population.

Trait	LG	LOD	% variation	*p*‐Value	Left end cM (LOD)	Highest peak cM (LOD)	Right end cM (LOD)
Upper lateral line	05	1.614	3.301	.029	2.0 (0.086)	67.4 (1.614)	73.3 (0.459)
	09	1.789	3.668	.019	0.0 (1.789)	0.0 (1.789)	44.1 (0.704)
	19	2.544	5.264	.004	10.0 (1.446)	23.5 (2.543)	38.0 (1.416)
	21	2.220	4.576	.008	12.0 (1.210)	27.0 (2.220)	51.0 (1.218)
Lower lateral line	19	2.254	4.810	.007	11.0 (1.185)	38.5 (2.254)	51.0 (1.151)
	22	2.492	5.332	.004	0.0 (1.858)	7.0 (2.492)	31.16 (1.247)
	24	1.774	3.763	.020	0.00 (1.474)	0.9 (1.774)	72.15 (0.098)
Caudal vertebrae	01	3.017	5.391	.001	13.0 (1.560)	30.00 (3.017)	36.5 (1.539)
	03	3.834	6.920	<.001	9.5 (1.879)	85.9 (3.834)	85.9 (3.834)
	14	1.529	2.682	.036	0.0 (1.000)	1.8 (1.529)	77.0 (0.166)
	22	3.672	6.615	<.001	23.0 (2.605)	31.2 (3.672)	41.5 (2.626)
	24	3.323	5.960	<.001	48.8 (1.832)	58.0 (3.323)	62.5 (1.889)
Abdominal vertebrae	19	1.387	3.165	.046	2.0 (0.786)	41.5 (1.387)	62.8 (0.836)
	22	0.981	2.227	.114	4.0 (0.091)	39.0 (0.981)	60.7 (0.923)
	24	1.352	3.083	.050	15.2 (0.893)	28.5 (1.352)	70.5 (0.643)
Total lateral line	09	1.558	3.092	.032	0.0 (1.558)	0.0 (1.558)	69.1 (0.160)
	19	3.282	6.653	<.001	12.5 (2.359)	26.5 (3.282)	40.0 (2.372)
	21	1.715	3.410	.023	14.5 (0.586)	40.5 (1.715)	59.0 (0.607)
	24	2.322	4.652	.006	0.0 (1.875)	0.9 (2.322)	72.2 (0.176)
Total vertebrae	03	3.307	5.905	<.001	7.0 (1.912)	85.9 (3.307)	85.9 (3.307)
	14	1.644	2.875	.028	0.0 (1.096)	1.8 (1.644)	77.8 (0.300)
	22	4.487	8.132	<.001	23.5 (3.503)	31.2 (4.487)	39.5 (3.502)
	24	4.386	7.938	<.001	53.5 (3.167)	58.0 (4.386)	62.0 (3.092)

*Note*: Linkage group (LG), logarithm of odds (LOD) scores, the percentage of trait variation explained, and *p*‐values for the quantitative trait loci (QTL) regions resulting from the drop one QTL at a time ANOVA test are given. The 95% Bayesian credibility interval position expressed in centiMorgans (cM) and LOD scores for each QTL peak are detailed for each trait assessed.

In terms of vertebrae counts, no significant (*p* ≥ .05) QTL was found for the relatively invariant abdominal vertebrae trait (Table 2), and this trait was subsequently excluded from Figure 5. In contrast, the model for the caudal vertebrae identified five QTLs, but only one with genome‐wide significance, collectively explaining 30.1% of the variance (Table 2, Figure 5c). Notably, three QTLs also reached genome‐wide significance for total vertebrae, accompanied by an additional QTL at the chromosome level, accounting for 28.5% of the total variation (Table 2, Figure 5e). Particularly, LGs 3, 14, 19, 22, and 24 were common across two or more of the examined meristic traits (Table 2). The LOD scores for LGs 22 and 24 were especially high, suggesting their potential importance for both the posterior lateral line system and the vertebrae numbers (Figure 5). Bayesian credibility intervals for the left and right ends of each QTL showed overlapping genomic regions in LGs 19, 22, and 24 among the upper and lower lateral line scales, as well as the caudal and abdominal vertebrae traits assessed here (Table 2).

Of the approximately 130 genes expressed in developing somites and lateral lines in African cichlids (Bloomquist et al., 2017), nearly 30 of these genes were found to lie under our QTL peaks based on the Midas genome annotation (Kautt et al., 2020). Several of these putative candidate loci are discussed.

## 4 DISCUSSION

Across a diversity of Neotropical Heroine cichlid species, there is a statistically supported positive phylogenetic correlation between the total lateral line scales and the total vertebrae number, as previously hypothesized by Říčan et al. (2016). However, the meristic values of the lateral line and vertebrae among the 13 species of recently diversified Nicaraguan Midas cichlids generally still overlapped. Nevertheless, dividing the number of lateral lines scales and vertebrae into their components: upper and lower, abdominal and caudal, respectively, did provide valuable insights into the anatomical divergence of Nicaraguan Midas species. We found a polygenic basis for the number of total lateral line scales and total vertebrae, and also identified two overlapping QTLs (Figure 3b,c on chromosomes 22 and 24) between the lateral line scales counts and the vertebrae numbers, suggesting a co‐localization of genetic factors responsible for their divergence. This suggests that the co‐evolution of these two traits might, at least in part, be due to a few genetic pathways that are biasing their largely independent evolution. These findings provide novel insights into potential genetic mechanism underlying Heroine cichlid meristic covariation.

The meristic ranges for the lateral line scales in our wild‐caught Midas specimens are consistent with prior reports of these cichlid species (Geiger et al., 2010; Recknagel et al., 2013; Říčan et al., 2016; Stauffer et al., 2008; Stauffer & McKaye, 2002). Although the lateral line scales for cichlids are most commonly reported in terms of their total number (Geiger et al., 2010; Recknagel et al., 2013; Říčan et al., 2016; Stauffer et al., 2008; Stauffer & McKaye, 2002), our findings indicate that splitting these meristic data into their upper and lower components could help in limited cases to distinguish several Midas species (Table 1). For example, the number of the upper lateral line scales between the benthic *A*. *citrinellus* from crater Lake Apoyeque and the limnetic *A*. *sagittae* endemic to the crater Lake Xiloá are statistically different. In addition, species such as *A*. *viridis* seem to have more upper lateral line scales (22.6) than *A*. *xiloaensis* (20.7), and both species are endemic to the Xiloa crater lake benthic microhabitats. Further, *A*. *xiloaensis* tends to have fewer scales than other Midas species, such as *A*. *globosus* (21.4) or *A*. *labiatus* (21.2), all of which are repeatedly evolved benthic species, although they are endemic to different lakes. The variation we observed in the upper and lower lateral lines in the Midas cichlids suggests these characters are likely highly variable in many recently diverged cichlids and should be more commonly employed when finely delineating cichlid species.

Vertebrae counts in cichlids should also be examined more widely. For example, Říčan et al. (2016) conducted a characterization of mostly riverine Heroine species, categorizing them into two ecomorphs: a fast‐flowing or lotic group with approximately 27 vertebrae and a slow‐flowing or lentic group with approximately 32 vertebrae. This ecological differentiation is mainly associated with body elongation and indicates the relationship between body elongation and internal structures such as vertebrae number, which may be the most common case but needs to be studied more extensively, including in the Nicaraguan Midas complex (Franchini et al., 2014; Fruciano et al., 2016; Hulsey et al., 2018; Kautt et al., 2020). Our results further emphasize that separating vertebrae into their functional categories, such as abdominal and caudal vertebrae, offers additional biological and taxonomical insights (Table 1). For example, the benthic species *A*. *globosus* has 15 abdominal vertebrae, distinguishing it from all other Nicaraguan Midas that generally have only 14 abdominal vertebrae. A similar pattern emerges for *A*. *viridis*, which often displays fewer caudal vertebrae than its sympatric congeners that co‐occur in crater Lake Xiloa (e.g., *A*. *amarillo*, *A*. *sagittae*, or *A*. *xiloaensis*). However, as has been recently proposed by Baxter et al. (2022), morphological and behavioral differences between limnetic and benthic species may encompass not only variation in vertebral counts but also other mechanical properties of the central axis, such as vertebrae stiffness or vertebrae bending ratio that influence caudal locomotion. There remains an extensive opportunity to examine postcranial diversification within cichlids including the young *Amphilophus* radiation examined here.

There are a number of potential genes associated with the development of the lateral line scales and vertebrae numbers that could provide candidate loci to examine more fully in the future Bloomquist et al. (2017). For example, the genes *pax6*, *cldn15a*, and *hoxa10* are found under our QTL on LG 01, 03, and 22. Similarly, the genes *sostdcl*, and *dlx5* are both expressed in developing lateral lines and somites and are present under our QTL peak on LG 22. Other putative candidate genes, such as *foxa3*, *lhx9*, *tp63*, and *notch2*, are located under our QTL peak on LG 24 and influence the development of the meristic traits studied here (Bloomquist et al., 2017). These loci could be explored further in the meristic divergence of Midas and other cichlids.

The genetic architecture of the lateral line system and scales has been explored in various fish species (Liu et al., 2014; Nichols et al., 2004; Peichel et al., 2001; Powers et al., 2020; Wark et al., 2012; Yang et al., 2016). In general, these authors reported a polygenic basis for lateral line scale numbers associated with several loci and QTL peaks. Our results align with these studies, as we found up to four loci that seem to contribute to the delimitation of lateral line scale traits assessed (Table 2). However, because our QTL analyses were based on a single parental cross, we may have not detected a number of independent and co‐segregating loci governing these meristic traits (Poore et al., 2023). Further, the low effect size of the QTL identified did not explain a substantial amount of meristic variation (Table 2). Therefore, the only partial co‐segregation of the identified QTL and the low magnitude of meristic variation explained in both traits could have had limited effects on their meristic co‐evolution (Poore et al., 2023). Yet, previous research conducted on the same laboratory‐reared F2 specimens examined here identified the genomic underpinnings of several traits such as body shape, caudal peduncle width, and pharyngeal jaw morphology (Franchini et al., 2014; Fruciano et al., 2016; Kautt et al., 2020), and generally recovered a polygenic basis for these other traits. Interestingly, LG 3 was found previously to have a strong statistically significant effect on body shape (Franchini et al., 2014; Fruciano et al., 2016; Kautt et al., 2020), and also seems to contribute based on our analysis to the caudal and total vertebrae numbers. In fact, Fruciano et al. (2016) identified the QTL on LG 3 as having the strongest effect on both the Midas pharyngeal jaw morphology and body shape. Additionally, QTLs for body shape were previously found on LG 5, 9, and 19 (Fruciano et al., 2016; Kautt et al., 2020), which were all LGs recovered in our QTL models for the lateral line scale numbers. Further, LG 14, associated with body shape in both Franchini et al. (2014) and Kautt et al. (2020) studies, was also implicated in our study for influencing caudal and total vertebrae numbers. Kautt et al. (2020) also isolated one of the two peaks for Midas lip size on LG 24, which was selected by our QTL models for a number of traits, including lower lateral line scales, total lateral lines scales, caudal vertebrae, and total vertebrae numbers. These concordant QTL peaks, observed on the same F2 individuals, suggest there could be a genomic relationship between body shape and several meristic traits that characterize the postcranial body axis. Co‐segregating genomic regions controlling trait divergence could often influence the co‐evolution and adaptive divergence of many rapidly radiating groups like cichlids fishes.

## AUTHOR CONTRIBUTIONS

**Nicolas Ehemann:** Conceptualization (equal); data curation (equal); formal analysis (equal); investigation (equal); methodology (equal); visualization (equal); writing – original draft (equal); writing – review and editing (equal). **Paolo Franchini:** Conceptualization (equal); formal analysis (equal); methodology (equal); supervision (equal); writing – review and editing (equal). **Axel Meyer:** Conceptualization (equal); funding acquisition (lead); investigation (equal); methodology (equal); project administration (lead); resources (lead); supervision (equal); validation (equal); writing – review and editing (equal). **C. Darrin Hulsey:** Conceptualization (equal); data curation (equal); formal analysis (equal); funding acquisition (equal); investigation (equal); methodology (equal); project administration (equal); resources (equal); supervision (equal); validation (equal); visualization (equal); writing – original draft (equal); writing – review and editing (equal).

## CONFLICT OF INTEREST STATEMENT

We have no competing interests to declare.

## Supporting information


Table S1.

Table S2.

## Data Availability

The datasets analyzed supporting the findings of this study are available in the supplementary files.
